# Malignant Melanoma Presenting as Spinal Cord and Pleural Lesions

**DOI:** 10.1155/2023/9647892

**Published:** 2023-02-21

**Authors:** Aysha Albastaki, Sharifa Ahmed, Asher Khan, Abeer Farhan, Talal Almayman

**Affiliations:** ^1^King Hamad University Hospital, Al Muharraq, Bahrain; ^2^Royal College of Surgeons-Medical University of Bahrain, Al Muharraq, Bahrain

## Abstract

Primary spinal cord melanoma (PSCM) and primary pleural melanoma (PPM) are extremely rare entities with scarce cases reported in the literature. We present a case of a 54-year-old male diagnosed with possible primary pleural melanoma and primary spinal melanoma, managed with partial surgical resection, postoperative radiotherapy, and chemotherapy consisting of Ipilimumab, nivolumab, and temozolomide. This leads to decreased symptoms and improved quality of life of the patient. In this case report, we review the literature on PSCM and PPM in detail, addressing the pertinent clinical aspects as well as current and upcoming therapeutic options.

## 1. Introduction

Malignant melanoma (MM) is the most aggressive form of skin cancer. In the recent years, major advances in diagnostic and therapeutic research have increased our understanding of malignant melanoma and led to the identification of new therapeutic targets and strategies, improving the prognosis of affected patients. [1] The most common origin for primary MM is the skin which accounts for 91.2% of all reported cases of primary MM [2]. Other primary sites for melanoma are relatively uncommon. Primary spinal cord and primary pleural melanomas are extremely rare malignancies, with only 9 reported cases of primary pleural melanomas in the literature and a relatively small number of cases of primary spinal cord melanomas [3, 4]. To the best of our knowledge, this is the first case of a melanoma presenting as a spinal cord and pleural lesion.

## 2. Case Presentation

A 54-year-old male patient presented to the emergency department with a two-month history of neck and back pain. The back pain was localized to the interscapular region, constant, and progressively worsening. No other associated symptoms were present such as upper and lower limb weakness or numbness, headaches, changes in vision, hearing, or taste, or changes in urinary or bowel habits. System review was otherwise negative. The patient did not have a history of similar symptoms. The patient was a known case of type 2 diabetes mellitus and has been well controlled on insulin and metformin (500 mg BD) for the last 10 years. The remainder of the patient's medical history was unremarkable; the patient works in an office job, has no history of malignancy in the family, and does not smoke nor drink alcohol.

**Figure 1:**
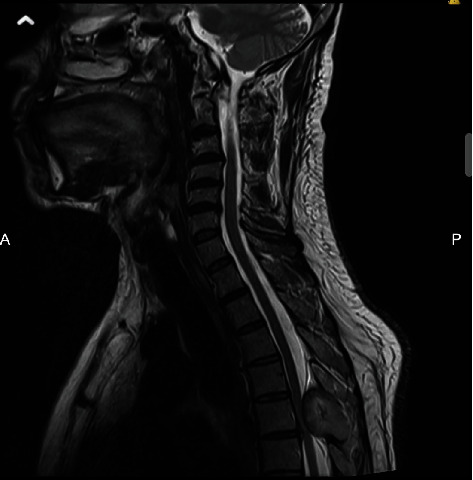
T2-weighted sagittal MR of the neck showing the spinal lesion at the level of T5.

**Figure 2:**
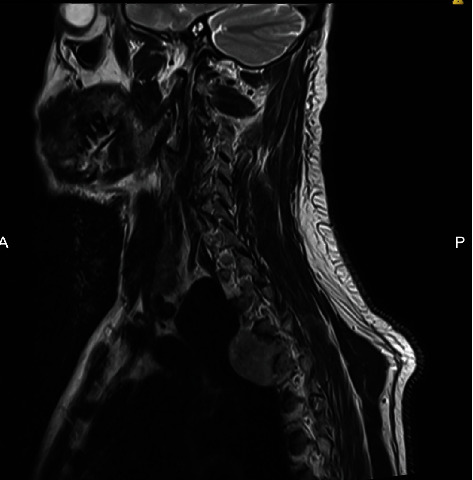
T2-weighted sagittal (view/section) MRI of the neck showing the pleural lesion.

On the 26th of January 2019, the patient went to a private clinic where an MRI of his dorsal spine revealed a large expansile lesion seen in the posterior element of the T5 vertebrae, involving both laminae, spinous processes, the facet joints, both pedicles, and the transverse processes. The lesion measured 2.5 × 4 × 3.2 cm in the anteroposterior (AP), transverse, and longitudinal axes, respectively. There was resorption of the spinal canal cortical outline, and the lesion was significantly encroaching upon the spinal canal, causing significant spinal cord compression. At the site of maximal compression, the spinal canal measured 3 mm compared to 1.5 cm at the T4 level superiorly. On T2-weighted MRI, the signal intensity of this lesion was heterogenous with a bright thick signal on the outer aspect of the lesion and low central signal intensity (Figure 1). On T1-weighted MRI, the signal intensity was inversed, brighter in the central part of the lesion and darker on the periphery. There is only minimal post-contrast enhancement which was mainly peripheral. Another well-defined, round lesion was noted on the right side at the T3-4 level, located retropleural, and paravertebral. The lesion had similar signal characteristics as the spinal lesion, along with areas of calcifications on contrast-enhanced CT. The lesion measured 3.7 × 3.4 × 3.4 cm in the AP, transverse, and longitudinal axes, respectively (Figure 2). Despite abutting on the right vertebral wall, there were no signal abnormalities on PET-CT within the vertebral body or adjacent bony structures. There was also no obvious intrathoracic infiltration. Thus, this lesion likely originates from the pleura.

## 3. Examination

On physical examination of the patient, there were no obvious structural deformities, the patient displayed a full range of motion in both the thoracic and lumbar spine, and the neurological examination of both upper and lower limbs was completely normal.

One day after admission, a contrast-enhanced computed tomography (CT) scan of the thoracic spine was conducted at our institution which confirmed the findings. Due to the compression of the spinal cord, the patient underwent an urgent decompressive laminectomy and resection of the dura followed by excision of the posterior column tumor at the T4-6 region under general anaesthesia on the 29th of January 2019. Intraoperatively, a highly vascular black mass was seen which was then debulked.

On the 5th of February 2019, the patient's histological report showed a malignant cellular neoplasm composed of sheets, nests, and clusters of pleomorphic malignant cells with hyperchromatic nuclei, prominent nucleoli, and eosinophilic to scanty cytoplasm. Moreover, abnormal pigment deposition, melanin, was seen in most of the cells. The tumor was positive for Melan-A, HMB-45, and S-100, as well as a positive Ki-67 expression of 96%, thus confirming the diagnosis of melanoma. The patient was discharged on the same day and received follow-up from neurosurgery, thoracic surgery, oncology, and endocrinology in the outpatient clinic. The patient had a full physical examination by ophthalmology, otolaryngology, and dermatology for any evidence of primary lesions on the skin, mucosa, or nails. No lesions were found. The patient was seen in the endocrinology clinic where it was observed that his fasting blood glucose level was well controlled, and he was discharged on only oral metformin with a dose of 500 mg twice daily.

**Figure 3:**
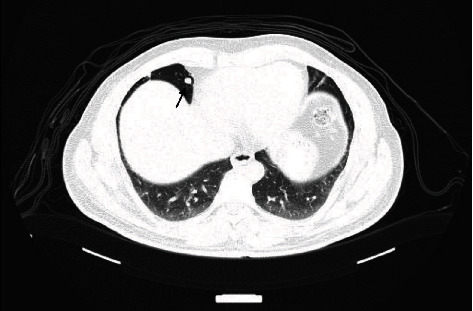
High-resolution chest CT shows a pulmonary nodule in the right middle lung lobe denoting pulmonary metastasis.

**Figure 4:**
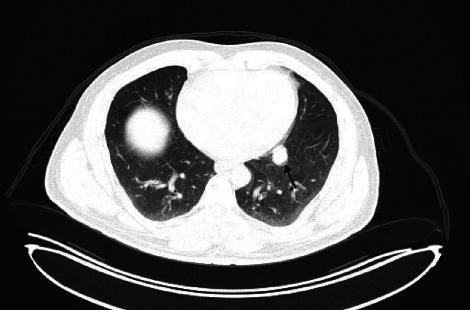
High-resolution chest CT showing another left lower lung lobe paracardiac lung mass denoting another metastatic lesion.

On the 11th of February 2019, a positron emission tomography-computed tomography (PET-CT) scan was conducted and showed that the mass at the T3-4 region was fluorodeoxyglucose (FDG) avid, with a maximum standardized uptake value (SUVmax) of 5.4. No other hypermetabolic lesions were detected in the rest of the surveyed body. Following surgery, the patient received palliative radiotherapy to the dorsal spine. The patient's management was continued using 9 cycles of nivolumab. Unfortunately, follow-up imaging on the 15th of July 2019 showed the development of a new metabolically active metastatic lesion in the right middle pulmonary lobe and left lower paracardiac lung lobe, and progression of the size and metabolic activity of the lesions denoting lung metastasis (Figures 3 and 4). Thus, the patient was started in ipilimumab in addition to nivolumab. The thoracic team was consulted and decided that total resection is not deemed feasible due to intraspinal extension of the primary lesion as well as multiple lung metastases.

Further follow-up imaging showed progression of the spinal lesion as well as lung nodules. Since then, the patient has been started on temozolomide. He completed 10 cycles of temozolomide at a dose of 270 mg once daily for 5 continuous days. During follow-up, imaging revealed metabolic regression of the spinal lesion as well as the pulmonary nodules. Clinically, he became symptomatically well with significant improvement of the back pain that he now describes as mild, intermittent, and adequately managed with paracetamol. As a result of this, the patient has been able to return to work.

## 4. Discussion

Malignant melanoma is considered the most fatal form of skin cancer. It represents less than 5% of all cutaneous malignancies, while accounting for the majority of skin cancer deaths [5, 6]. Most commonly, malignant melanoma is present on the skin, and lesions discovered in other organs such as the CNS and the pleura are secondary to metastasis. PSCM are an even rarer occurrence, accounting for approximately 1% of melanoma cases [7–9] or 0.005 cases per 100,000 [10]. The peak incidence is in the fifth decade of life, with no apparent predilection towards the male or female sex [7]. PSM are located either intradurally or extradurally and may possess intra- or extramural components [11]. A review of 60 cases of PSM by Li et al. [12] showed that 62.4% were extramedullary while 37.7% were intramedullary. There are also reports of multifocal primary cervical spinal cord melanomas. With regard to the distribution along the spinal cord, the lesions are most located in the thoracic, cervical, and lumbar regions respectively [12–15].

### 4.1. Clinical Presentation of Spinal and Pleural Melanomas

Multiple reports of CNS melanoma in the literature have shown different clinical presentations including symptoms of myelopathy, dysesthesia, weakness, pain, and fecal and urinary incontinence [13, 15–17]. Hydrocephalus has also been reported as spinal cord lesions that can disturb the cerebrospinal fluid circulation [18]. In the case of pleural melanomas, the main symptoms include dyspnea, chest pain, and cough [3].

### 4.2. The Origin of Primary Pleural and Spinal Cord Melanomas

Primary CNS melanomas may arise from either neuroectodermal rest cells or leptomeningeal melanoblasts, which originate from the neural crest [7–9, 19]. A hypothesis for development of primary melanoma suggests that a few neural crest cells do not migrate during embryogenesis and stay within the neural tube [20]. These cells fail to establish the signaling pathways required to normally differentiate and mature. Moreover, several hypotheses have been proposed regarding the development of a primary melanoma from areas devoid of melanocytes, such as in the pleura. Hypotheses include growth from pigment blast cells, growth from multipotent stem cells, squamous metaplasia, growth from aberrant skin nevus cells which transfer along the lymphatic pathway, or, alternatively, disappearance of the primary tumor after metastasis [21].

### 4.3. Diagnosis of Spinal and Pleural Melanomas

Early and correct diagnosis is the key for ensuring the best clinical outcome. MRI remains the gold standard for diagnosing spinal and pleural melanomas. Characteristic features on MRI include hyperintensity on T1-weighted sequencing, hypointensity or isointensity on T2-weighted sequences, and homogenous enhancement with gadolinium contrast agents [7, 15, 22]. The paramagnetic properties of melanin or hemorrhagic elements in the tumors are reported to be the reason behind these signal intensity features [7]. Therefore, MRI findings vary depending on the number of melanin-containing cells and the presence or absence of hemorrhage [14]. These characteristics are similar to other tumors of melanocytic origin. Although MRI imaging aids in the diagnosis, it does not differentiate between primary melanoma and other malignant lesions. This can be done by exclusion of primary lesions via evaluation by ophthalmology, otolaryngology, and dermatology. CT findings include a hyperdense appearance of the lesion when enhanced by intravenous contrast [23].

The definitive diagnosis of melanoma can only be made by histopathological examination of an excisional biopsy [24]. Histopathological features include spindle or epithelioid cells arranged in sheets, nests, or whorls with well-differentiated melanocytes and melanin-rich cytoplasm [25]. Markers on immunohistochemistry include HMB-45 and S-100. Moreover, as molecular mechanisms are better understood, researchers and clinicians have been able to utilize molecular biomarkers and immunohistochemistry in the interpretation of difficult cases [26]. Immunohistochemistry is now the most commonly used technique by pathologists to diagnose melanoma and is reliable, inexpensive, and relatively available. Some of the melanocytic biomarkers with highest specificity include Melan-A, MART-1, and HMB-45, the expression of which is mostly limited to melanocytic tumors. The most commonly used proliferation marker is Ki-67 which is elevated in most highly aggressive melanomas, such as in our patient [26].

Upon histopathological diagnosis, the next step is to determine if the lesion is primary or metastatic. This is done by a thorough and complete examination of the skin, including inspection of the mucosa and genitalia. Moreover, ophthalmological (including retinal) and gastrointestinal examination is also necessary [7, 27]. PET-CT following FDG injection is accurate in detecting additional foci of disease [9, 27]. The FDG PET-CT can show the difference between malignant and benign tumors, where the pleural malignant tumor may show focal or diffuse increased FDG uptake with standardized uptake values (SUV) of 10 or greater. However, the increase of FDG uptake is mild and in pleural benign diseases with SUV approximately equal to 1. FDG may be also used to help localize the site for biopsy and improve accuracy [3].

It is difficult to differentiate between various melanin-containing tumors intraoperatively; however, features such as dural mater attachment and a distinct dark black color may indicate that the tumor originates from leptomeningeal melanocytes [12]. However, surgeons need to keep in mind that spinal meningiomas may mimic this appearance when complicated by hemorrhage [12].

### 4.4. Staging

Aside from the Clark and Breslow's depth used to stage cutaneous melanoma, the American Joint Committee on Cancer (AJCC) proposed the TNM (tumor, node, and metastasis) staging system. It provides a guideline for staging a patient by combining the histological attributes of the primary tumor (T), the presence and extent of regional lymph node disease (N), and the presence and extent of metastases (M). The guideline was most recently revised in 2018 [28].

### 4.5. Medical and Surgical Management

The management of melanoma is a complex and continually evolving subject. Plastic surgeons, dermatologists, oncologists, surgical oncologists, and neurosurgeons must keep up to date with recent changes in the field. There have been several new and innovative drugs developed over the last 10 years that have greatly improved the prognosis for patients with metastatic melanoma secondary to the identification of new biomarkers and drug targets. For stage IV (or metastatic) melanoma, the National Comprehensive Cancer Network recommends Ipilimumab (IgG1 anti-CTLA-4 antibodies), vemurafenib (BRAF inhibitors specific to melanoma harboring the BRAF V600E and V600E/K mutations), dabrafenib, and high-dose interleukin-2 as first-line agents. Moreover, other newly emerging therapies include the anti-programmed cell death 1 receptor agents (nivolumab and pembrolizumab) and oncolytic vaccines. The most well-known of the immunotherapies targeted against cytotoxic T lymphocyte-associated antigen 4 (CTLA-4) and programmed cell death protein 1 (PD-1) have become the frontline treatment of metastatic melanoma in many clinical scenarios [29]. Ipilimumab, which was used in our case, has (been shown to be beneficial) shown benefit in patients with metastatic melanoma in multiple clinical trials. Moreover, a phase 3 clinical trial is currently ongoing/underway comparing nivolumab, ipilimumab, and a combination of the two (NCT01844505). Combination therapies involving the use of multiple immune modulating agents show great promise and may be commonplace in future treatment plans [30].

For spinal cord melanomas, complete surgical resection is the treatment of choice [7, 15, 31]. However, some reports show long-term survival even with subtotal resection [14]. Additionally, adjuvant radiotherapy is recommended in all cases [7, 15, 16, 31]. In literature, the dose of radiation administered ranged between 30 and 60 Gy, with the mean total dose found to be 47 Gy [14]. The use of chemotherapy also seems to be promising especially in cases with incomplete resection [14, 31]. Studies have shown that temozolomide, as used in our patient, although costlier than other chemotherapeutic agents, is well tolerated and has the benefit of the additional advantage of improving the quality of life of patients with metastatic melanoma [32, 33].

A meta-analysis showed that chemotherapy and biological therapy were capable of reducing the recurrence rate and increasing survival (rates) only by 3% after five years [12, 34].

Although immunotherapy is the current focus of interest, there is no literature available regarding the use of immunotherapy in PSCM or PPM.

### 4.6. Follow-Up and Surveillance

Surveillance imaging every 6 months is supported by very limited data, and some patients with high-risk and aggressive disease require more frequent imaging (for instance, every 3 months). Yet, serial imaging is recommended at regular intervals even after complete surgical resection [19]. Moreover, follow-up of patients with melanoma, regardless of radiological surveillance, should include regular routine clinical examinations as well as a full skin examination and palpation of nodal basins [35].

### 4.7. Prognosis

Primary CNS melanoma has a much better prognosis compared to metastatic CNS melanoma (which has median survival between 3 to 6 months) [11, 14, 31]. A proposed explanation (for this) is due to the lack of lymphatic vessels in the CNS, limiting potential for metastasis [14]. The clinical course and survival are unpredictable of primary CNS melanoma, with some cases reporting several years of survival [15], whereas others reporting months [14]. Larson et al. reported an average life expectancy of approximately 7 years after surgery with radiotherapy [17]. In a review done by Kim et al. [14], the mean duration of survival was 28.8 months and with a range of 3 months to 13 years. In one case report, the progression of the tumor was controlled for 21 years. This may have been due to radiation therapy as well as the intrathecal injection of interferon beta [36]. Metastasis was observed in patients after surgery, and sites of metastases include the brain, lung, bone, and other systemic metastases [11, 37, 38].

## 5. Conclusion

In summary, we report a rare case of metastatic malignant melanoma presenting as a spinal and pleural lesion. The case highlights the rarity of such presentation. We review the available literature on this rare presentation and the recommended available medical and surgical treatments, as well as upcoming therapeutic options.
